# Effect of Lead Exposure and Ergonomic Stressors on Peripheral Nerve Function

**DOI:** 10.1289/ehp.8106

**Published:** 2005-08-08

**Authors:** Margit L. Bleecker, D. Patrick Ford, Christopher G. Vaughan, Karen N. Lindgren, Michael J. Tiburzi, Karin Scheetz Walsh

**Affiliations:** Center for Occupational and Environmental Neurology, Baltimore, Maryland, USA

**Keywords:** bone lead, cumulative lead dose, ergonomic stressors, lead dose thresholds for peripheral nerve, peripheral nerve fiber size

## Abstract

In this study we investigated the effect of recent and chronic lead exposure, and its interaction with ergonomic stressors, on peripheral nerve function. In a cross-sectional design, we used retrospective exposure data on 74 primary lead smelter workers. We measured blood and bone lead levels and, from historical records, calculated lead dose metrics reflecting cumulative lead exposure: working-lifetime integrated blood lead (IBL) and working-lifetime weighted-average blood lead (TWA). We additionally created five metrics related to IBL that cumulated exposure only above increasing blood lead levels ranging from 20 to 60 μg/dL (IBL20–IBL60). Current perception threshold (CPT) assessed large myelinated (CPT_2000_), small myelinated (CPT_250_), and unmyelinated (CPT_5_) sensory nerve fibers. Using multiple linear regression, we modeled CPT on the different measures of lead dose after adjusting for relevant covariates. CPT had a curvilinear relationship with TWA, with a minimum at a TWA of 28 μg/dL. Both TWA and IBL accounted for a significant percentage of the variance of CPT_2000_ (Δ*R*^2^ = 8.7% and 3.9%, respectively). As the criterion blood lead level increased from IBL20 through IBL60, so did the percentage of CPT_2000_ variance explained, with Δ*R*^2^ ranging from 5.8% (*p* < 0.03) for IBL20 to 23.3% (*p* < 0.00) for IBL60. IBL60 also significantly contributed to the explanation of variance of CPT_250_ and significantly interacted with ergonomic stressors. Measures of chronic blood lead exposure are associated with impairment of large and small myelinated sensory nerve fibers. This effect is enhanced at the highest doses by ergonomic stressors.

The classic description of lead neuropathy is that of a motor neuropathy that typically presents as wrist drop. More recently, investigators demonstrated that, in the development of lead neuropathy, sensory nerve fibers are affected earlier than motor nerve fibers (Chuang et al. 2000; Kovala et al. 1997; Rubens et al. 2001; Schwartz et al. 2001; Singer et al. 1983), and nerve conduction studies showed mild slowing of both sensory and motor conduction velocities as well as diminished amplitude of the sensory potential (Araki et al. 1986; Ashby 1980; Baker et al. 1984; Bordo et al. 1982; Buchthal and Behse 1979; Catton et al. 1970; Chen et al. 1985; Chia et al. 1996a, 1996b; Jeyaratnam et al. 1985; Kovala et al. 1997; Pasternak et al. 1989; Rubens et al. 2001; Seppäläinen and Hernberg 1980; Seppäläinen et al. 1979; Singer et al. 1983; Yeh et al. 1995). After reviewing the lead neuropathy literature from 1974 to 1984, Ehle (1986) concluded that sensory nerve conduction is more likely to be affected than is motor nerve conduction, that the upper extremities are more likely to be involved than the lower extremities, and that these effects usually occur after a year of lead exposure, with a continuous linear relationship between blood lead and nerve conduction velocity only when blood lead exceeded 70 μg/dL.

In evaluating peripheral nerve function, electrodiagnostic testing examines the integrity of only large myelinated nerve fibers with the fastest conduction velocities. Current perception threshold (CPT), a neuroselective test, measures sensory nerve conduction threshold in three nerve fiber populations—large myelinated (Aβ), small myelinated (Aδ), and unmyelinated (C) nerve fibers. In peripheral neuropathies associated with a variety of medical conditions, CPT abnormalities have demonstrated good agreement with nerve conduction studies (Katims et al. 1989; Rendell et al. 1989; Weseley et al. 1989). Additionally, pathology in the small myelinated and unmyelinated nerve fibers shown with CPT but not detected by routine nerve conduction studies occur in Fabry’s disease (Ro et al. 1999), diabetic and alcoholic C-fiber neuropathies (Oishi et al. 2002), arsenic exposure (Tseng 2003), and leprosy (Katims J, personnel communication). Capsaicin, a topical drug for pain relief that affects small nerve fibers, was found to elevate CPT thresholds for small myelinated and unmyelinated nerve fibers but not for large myelinated nerve fibers (Kiso et al. 2001).

In the past, the usual biomarker used to study lead neuropathy was PbB, a blood lead measure of recent exposure (Bordo et al. 1982; Davis and Svendsgaard 1990, Pasternak et al. 1989; Rubens et al. 2001). More recently, studies have shown an association between several biomarkers of chronic lead exposure—working lifetime-weighted average blood lead (TWA), working lifetime-integrated blood lead (IBL), and bone lead (PbBn)—and impairment of peripheral nerve function at a time when concurrent PbB was not elevated (Chia et al. 1996a; Chuang et al. 2000; Kovala et al. 1997; Schwartz et al. 2001; Triebig et al. 1984; Yeh et al. 1995). Which of these is the best metric for modeling chronic lead effects on the peripheral nerve remains to be demonstrated.

In the older literature, lead poisoning presented as muscle paralysis, typically occurring in the muscles most used (Aub et al. 1925). In fact, patterns of weakness differed by occupation but did not necessarily follow the distribution of a specific nerve (Cantarow and Trumper 1944). Although it is established that lead impairs peripheral nerve function, not studied to date is the effect of the interaction between lead exposure and chronic repetitive muscle use on that function.

We report here on the use of CPT to examine different nerve fiber populations in the upper extremities of a group of current lead workers with substantial chronic lead exposure and a broad range of ergonomic stressors (ESs).

## Materials and Methods

### Subjects.

A screening neuropsychological battery had been administered to 468 current and retired smelter workers by testers blinded to the degree of lead exposure of the worker. If performance on two or more tests in any functional domain was < 1.5 SDs compared with age-matched norms, the worker was invited for a complete clinical evaluation. Eighty current workers were identified by this criterion. Bleecker et al. (1995, 1997, 2002, 2003) and Lindgren et al. (1996) have described other aspects of these samples in previous publications. All participants volunteered for the study and signed an informed consent form approved by a combined provincial management–labor oversight committee. The Human Subjects Committee at the University of Maryland, Baltimore, approved the PbBn protocol.

### Exposure.

As employees of a primary smelter (located in New Brunswick, Canada), participants were routinely exposed to several sources of inorganic lead dust and, to a lesser extent, lead fumes. Since the smelter began operations in 1966, PbB levels of all employees have been checked at least quarterly. The frequency of PbB measurements depended on the relative degree of lead exposure of any given job and whether the employee had been relocated because of lead exposure. PbB levels precipitating relocation dropped from 90 μg/dL in 1966 to 75 μg/dL in 1974, 65 μg/dL in 1987, and 50 μg/dL in 1990. In general, the smelter workers in this study had chronic inorganic lead exposure that had been high in the distant past but lower in the more proximate past, with relatively low PbB levels at the time of this study.

Blood samples for lead testing had been collected preshift by the facility nursing staff in the infirmary, a building physically distinct from the smelter, using standard techniques to minimize the likelihood of lead contamination of the samples. A local off-site laboratory using the dithizone method initially performed sample analysis. By the early 1970s, these analyses were conducted by a regional contract laboratory using graphite-furnace atomic-absorption spectrophotometry; this laboratory subsequently became a participant in the interlaboratory blood lead proficiency testing program of the then–U.S. Centers for Disease Control. Results of this proficiency testing showed good agreement. For the purpose of this study, blood lead results from the two different laboratories were considered equivalent.

**Figure 1:**
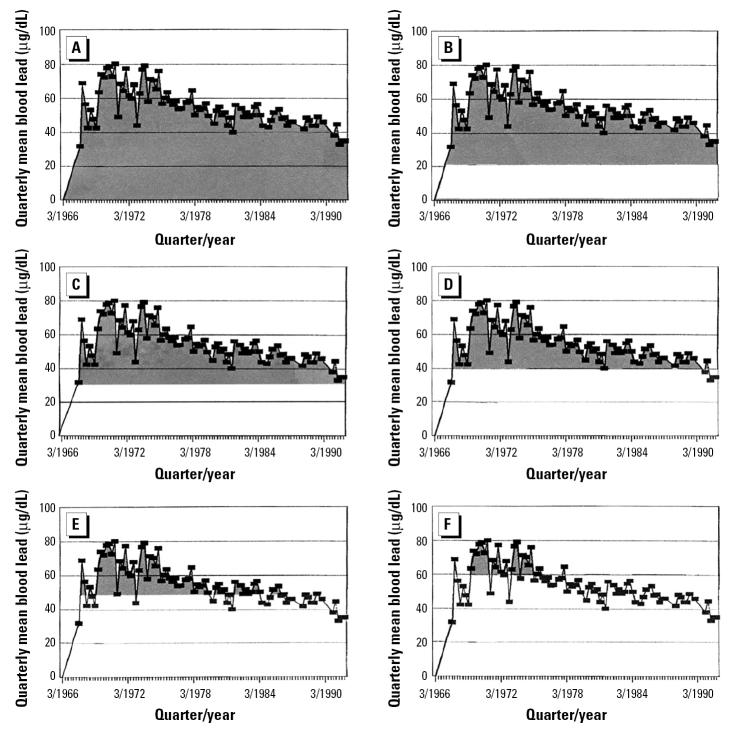
IBL above increasing criterion PbB levels, as shown by the shaded area under the curve. (*A*) IBL; (*B*) IBL20; (*C*) IBL30; (*D*) IBL40; (*E*) IBL50; (*F*) IBL60.

We calculated the lead levels used to determine IBL, a measure of cumulative blood lead, as the sum—over each participant’s working lifetime—of the products of each PbB level and one-half the time interval from the preceding blood lead to the following blood lead measure. TWA, the measure of average intensity of lead exposure over the period of employment, was created by dividing IBL by total years of employment at the smelter. To examine the effect of the amount of time a subject’s blood lead concentration was above a criterion level, we also created a series of metrics—IBL20, IBL30, IBL40, IBL50, IBL60—calculated in the same manner as IBL but including only areas under the time–blood lead curve that were above increasingly higher criterion blood lead levels; for example, IBL20 μg/dL was calculated by cumulating only that part of the area under the curve > 20 μg/dL (Figure 1). PbB was obtained on the day of testing. PbBn analysis, previously described (Bleecker et al. 1995), used the methods of Chettle et al. (1991). Measurements made at the mid-tibia with K-shell X-ray fluorescence were performed at the University of Maryland Toxicology Program laboratories.

### Working-lifetime weighted-average ES.

An ES rating was created with the assistance of the smelter safety committee, who reviewed all jobs ever worked by the participants and stratified them on a three-tiered ordinal scale. Using the method of Moore and Garg (1995), we converted the ordinal scale to interval with the following weights: 1, light; 6, medium; and 18, heavy. We then cumulated over each participant’s employment history the products of duration of time worked in a given job and the job’s assigned ES weight. From this, we calculated a time-weighted average ES.

### Current perception threshold.

CPT measures the minimum transcutaneous current intensity needed to produce a sensation (Neurometer, Neurotron Inc., Baltimore, MD). Because it uses a constant alternating current, there is no change in current intensity with variations in skin impedance. The sinusoidal waveform of the alternating current excites different subpopulations of nerve fibers as a function of frequency: 2,000 Hz, large myelinated fibers; 250 Hz, small myelinated fibers; and 5 Hz, small unmyelinated fibers.

Electrodes were attached to the dorsolateral aspect of the fourth digit of the nondominant hand. CPT was initially approximated by the “method of limits,” where the current was increased until the worker reported a sensation (i.e., buzzing). To more precisely ascertain threshold, the current was decremented and reincremented until a range was reached where a stimulus was correctly identified at one intensity and not at a slightly lower one for three consecutive trials. During this part of the testing, the stimulus presentation used a “forced choice method” paradigm with the presentation of a real and placebo stimuli. The procedure was repeated for all three frequencies at each site and are referred to in this article as CPT_2000_, CPT_250_, and CPT_5_.

### Data analyses.

Before the analyses, we examined age, current alcohol use, current smoking, ES, and the lead exposure metrics using univariate descriptive statistics to check for accuracy of data entry, missing values, and assumptions underlying multivariate analysis. Four individuals had values > 2.5 SDs above the mean of the CPT score and considered univariate outliers; one individual was identified through Mahalanobis distance as a multivariable outlier with *p* < 0.001. One individual was missing ergonomic data, leaving 74 individuals for analysis. Those removed were not significantly different from the remaining sample on the independent variables or the covariates.

SPSS-PC (version 12.0.1; SPSS Inc., Chicago, IL) was used for data analyses. The determination of covariates was based on risk factors associated with the development of a peripheral neuropathy. These included age, dichotomous current smoking, dichotomous current alcohol use, and working-lifetime weighted-average ES. Other medical conditions commonly associated with peripheral neuropathy were not present. The three CPT measures were modeled using multiple linear regression with the measures of lead dose, PbB, TWA, IBL, IBL20, IBL30, IBL40, IBL50, IBL60, and PbBn after adjusting for the covariates. Additionally, on the basis of *a priori* considerations (Hopkins and Morgan-Hughes 1969; Jacobs and Le Quesne 1984), we modeled the interaction between ES and each of the exposure variables in these regressions.

## Results

Demographic data for the 74 workers included in the analyses are presented in Table 1, along with mean values for the four measures of lead exposure and the outcome measures of CPT by frequency. As expected, thresholds by fiber population decreased from large myelinated nerve fibers to small myelinated nerve fibers and more so for small unmyelinated nerve fibers.

**Figure 2:**
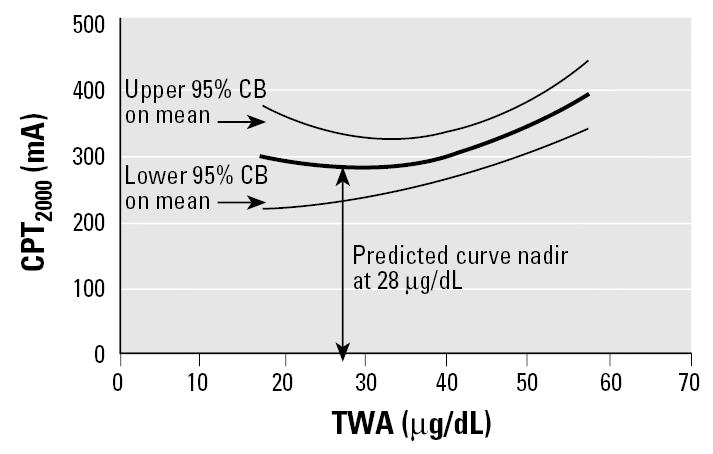
Predicted curvilinear relationship between TWA and CPT_2000_. CB, confidence bound.

Table 2 presents the results of the unique variance contributed to CPT by the measure of lead dose after adjustment for the covariates. Of the simple exposure variables, IBL, TWA, PbB, and PbBn, only the two based on cumulative blood lead levels—IBL and TWA—were significantly related to CPT, and in both cases only to CPT_2000_, after adjusting for the covariates. IBL explained 3.9% of the variation in CPT_2000_ (*p* < 0.08). Regression diagnostics revealed nonlinearity in the relationship between TWA and CPT_2000_, which was addressed by including a quadratic term in the model. Combined, the TWA and TWA^2^ terms accounted for 8.7% of the variation in CPT_2000_ (*p* < 0.03). The calculated minimum for the quadratic relationship for TWA and CPT_2000_ was 28 μg/dL (Figure 2).

To examine the contribution to CPT by exposure above different blood lead levels, we stratified IBL by the cumulative time a subject’s PbB was above different criterion levels—IBL above a PbB level of 20 μg/dL (*n* = 74), 30 μg/dL (*n* = 73), 40 μg/dL (*n* = 70), 50 μg/dL (*n* = 68), and 60 μg/dL (*n* = 61). The different sample sizes at each level reflect workers who did not have PbB that reached the required level. In Table 3, separate linear regressions revealed the unique variance that IBL20, IBL30, IBL40, IBL50, and IBL60 each contributed to the three frequencies of CPT, after adjusting for age, smoking, alcohol use, and ES. As the criterion PbB level increased from IBL20 through IBL60, so did the percentage of CPT_2000_ variance explained, with Δ*R*^2^ ranging from 5.8% (*p* < 0.03) for IBL20 to 23.3% (*p* < 0.00) for IBL60. Only IBL60 accounted for a significant amount of variance of CPT_250_, reflecting increased nerve damage with time spent at PbB > 60 μg/dL. Despite diminished power with IBL60 due to a smaller sample size, the dose effect remained significant.

**Figure 3:**
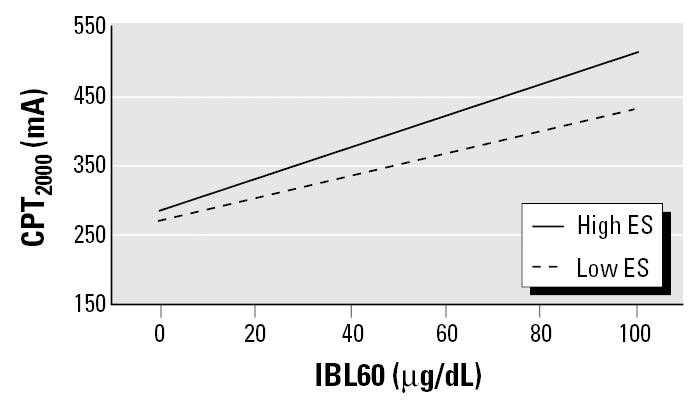
IBL60 and ES interaction.

To address the interaction of motor activity and lead toxicity on the peripheral nerves, we tested interaction terms created by multiplying the IBL variables based on the increased criterion blood lead levels × ES with multiple linear regression, controlling for the covariates and base terms. The strength of association of the interaction term with CPT_2000_ increased from IBL20 × ES (*R*^2^ = 1.1%, *p* = not significant) to IBL60 × ES (*R*^2^ = 6.1%, *p* < 0.02). The interaction is shown in Figure 3 as heterogeneity of regression slopes in the two groups stratified by high and low ES, suggesting that in the presence of high ES there is an enhanced lead effect on the peripheral nerve.

## Discussion

In this group of lead-exposed workers, IBL and TWA, two measures of chronic lead exposure, were significantly related to decrements in peripheral nerve function as measured by CPT, whereas PbBn and PbB were not. PbBn, with a half-life of 17–25 years, is a measure of lead stored in the bone compartment and is not a consistent biomarker of lead effect in the nervous system (Bleecker et al. 1997; Hanninen et al. 1998; Kovala et al. 1997). Also, PbB, with a half-life of 30 days, is a weak measure of lead exposure for the peripheral nervous system as demonstrated in a meta-analysis of 32 electrodiagnostic studies of lead neuropathy (Davis and Svendsgaard 1990). With ongoing exposure, lead accumulates in the nervous system and is retained there even as PbB falls. This accounts for the lack of a consistent relationship between lead content in the nervous system and PbB (Cantarow and Trumper 1944; Feldman et al. 1977; Goldstein et al. 1974). Because lead neuropathy requires exposure for months to years, it is not surprising that PbB, a biomarker reflecting recent exposure, has an inconsistent association with this outcome. Other studies have found measures of chronic lead exposure associated with changes in nerve conduction velocity at a time when PbB was not (Chia et al. 1996a, 1996b). However, measures of chronic lead exposure associated with vibration thresholds or nerve conduction studies continue to vary among the published studies, from PbBn (Schwartz et al. 2001) to TWA (Chuang et al. 2000; Seppäläinen et al. 1979; Triebig et al. 1984) to IBL (Chia et al. 1996a, 1996b; Kovala et al. 1997; Yeh et al. 1995). A Finnish study (Kovala et al. 1997) demonstrated that IBL had a stronger relationship than did PbBn with nerve conduction studies, a finding similar to that of this study.

The strength of IBL as a measure of cumulative exposure improved when the amount of time at lower blood lead levels was not included in the exposure term. This resulted in an increased strength of the linear model from Δ*R*^2^ for IBL = 3.9% (*p* < 0.08) to Δ*R*^2^ for IBL20 = 5.8% (*p* < 0.03). One possible explanation is that blood lead levels less relevant to the outcome were removed. This would result in improved precision of measurement due to a decreased nondifferential exposure misclassification.

IBL is a term composed of duration and intensity of exposure; however, the mean duration of lead exposure in the literature reporting significant association between IBL and peripheral nerve conduction parameters varies from 2.5 years (Yeh et al. 1995), to 5.3 years (Chia et al. 1996a, 1996b), to 16 years (Kovala et al. 1997), to 20 years in the present study with CPT. Despite decreased duration spent at the increasing criterion blood lead level, the variance accounted for by the exposure term increased, suggesting that average intensity may be more critical than duration of exposure for neurotoxicity in the peripheral nerves. In the present study, the absence of a significant relationship between years employed and CPT_2000_ is consistent with this hypothesis.

As reported by Ehle (1986), an association of nerve conduction studies with lead exposure occurred when PbB exceeded 70 μg/dL; however, an increasing number of studies are finding this association at much lower PbB levels. Chuang et al. (2000) found a threshold curve for vibration perception at a mean PbB level of 31 μg/dL. Chia et al. (1996a, 1996b) and Chen et al. (1985) suggested that the threshold effect for changes in nerve conduction studies occurs at a PbB level of 40 μg/dL, whereas Seppäläinen et al. (1983) showed that it was closer to 30 μg/dL. Yeh et al. (1995) found electromyographic abnormalities beginning at PbB levels of 17 μg/dL. In this study, there was no association of CPT and PbB; however, the curve minimum of TWA was at 28 μg/dL. This association of low PbB with nerve function may be caused by the attention given sensory nerve fibers that are affected earlier in the development of lead neuropathy (Ehle 1986; Rubens et al. 2001; Singer et al. 1983).

CPT for large myelinated fibers showed that these were the primary nerve fibers affected by lead exposure. Vibration perception thresholds also carried by large myelinated fibers is associated with chronic lead exposure (Chuang et al. 2000; Kovala et al. 1997; Schwartz et al. 2001). These findings agree with neuropathology of a biopsy of human lead neuropathy that found loss of the large myelinated nerve fibers in a sensory nerve (Buchthal and Behse 1979). CPT provided neuroselective stimuli that allowed for detection of expanded pathology at IBL60 with involvement of large (CPT_2000_) and small (CPT_250_) myelinated nerve fibers, a biologically plausible finding.

Lead affects the upper extremities more frequently than the lower extremities (Chuang et al. 2000; Ehle 1986; Pasternak et al. 1989; Schwartz et al. 2001; Yeh et al. 1995). Dermal absorption of inorganic lead is minimal compared with inhalation and oral absorption. However, direct cutaneous exposure in the upper extremities may occur through skin absorption, as reported in humans with limited exposure in an experimental setting (Moore et al. 1980; Stauber et al. 1994; Sun et al. 2002). This may contribute to the increased prevalence of upper-extremity involvement, because lower extremities are usually protected from cutaneous exposure. The upper-extremity involvement is unusual because toxic neuropathies classically begin in the largest and longest axons in the feet. Earlier literature of lead neuropathy reported different patterns of weakness in the upper extremities based on occupation, which some believed was due to a myopathy (Aub et al. 1925; Cantarow and Trumper 1944; Hamilton 1925). The conclusion reached was that motor activity increased the effects of lead toxicity (Jacobs and Le Quesne 1984). In the present study, exposure to ES, used as a surrogate for active motor units, did interact with lead exposure but was significant only at IBL60. This is not unexpected because the earlier literature usually reported motor involvement presenting as weakness or paralysis only at PbB levels > 60 μg/dL.

Another possible explanation for the interaction of lead and active motor units is that nerves affected by lead are more susceptible to traction or mechanical compression, as would occur in the carpal tunnel of workers with exposure to ESs such as heavy lifting and shoveling. This interaction between a peripheral neuropathy and a focal entrapment neuropathy exists in patients with diabetes (Gilliatt and Willison 1962), Guillain-Barré syndrome (Lambert and Mulder 1964), and familial neuropathy (Earl et al. 1964). This paradigm examined in animal models revealed that the onset of compression neuropathy in healthy animals took several months versus a few weeks in animals with an underlying neuropathy; this latter compression lesion was more severe (Hopkins and Morgan-Hughes 1969). Serial electrodiagnostic studies on the upper extremities of lead-exposed workers showed that the median nerve was more susceptible to the effects of lead than was the ulnar nerve (Chia et al. 1996b). This finding may again reflect the interaction with ES. The principle of increased susceptibility of a compromised peripheral nerve to a second insult is well known in oncology, where patients with preexisting neuropathy may develop incapacitating toxic neuropathies after the administration of safe doses of chemotherapeutic agents (Chaudhry et al. 2003).

The ability to infer a causal relationship between lead exposure and peripheral nerve function is limited in a cross-sectional study. IBL and TWA were based on blood lead levels obtained over the working lifetime of the participants, thus increasing the likelihood of any causal inferences made.

In this population of lead smelter workers, nerve function as measured by CPT is associated with impairment in large and small myelinated sensory nerve fibers with a threshold effect at a TWA of 28 μg/dL. Peripheral nerve impairment is associated with markers of chronic lead exposure, TWA and IBL, but not PbBn, and may be present when recent PbB is at an acceptable concentration. Even with chronic lead exposure, intensity is more important than duration of exposure. At higher levels of lead exposure, nerve fibers affected by lead are more susceptible to the presence of more active motor units as reflected by ESs.

## Figures and Tables

**Table 1:** Descriptive statistics on demographics, exposure, and finger CPT for 74 current smelter workers.

Variable	Mean ± SD	Minimum–maximum
Age (years)	44 ± 8.4	24 to 64
Education (years)	8 ± 2.8	0 to 13
Years employed	20 ± 5.3	4 to 26
Current alcohol users (%)	60	—
Current smokers (%)	14	—
PbB (μg Pb/dL)	26 ± 7.1	13 to 43
IBL (μg-year/dL)	891 ± 298.8	81 to 1,376
TWA (μg Pb/dL)	42 ± 8.4	17 to 57
PbBn (μg Pb/g bone mineral)	40 ± 23.8	−12 to 90
CPT_2000_ Hz (mA)	330 ± 72.4	180 to 512
CPT_250_ Hz (mA)	134 ± 50.5	32 to 278
CPT_5_ Hz (mA)	83 ± 37.9	16 to 190

Values are mean ± SD except where noted.

**Table 2:** Unique variance (%) of CPT in the finger explained by measures of lead dose.

Variable	CPT_2000_	CPT_250_	CPT_5_
IBL (μg-year/dL)	3.9*	0.4	0.0
TWA (μg Pb/dL)	—	0.8	0.3
TWA + TWA^2^ (μg Pb/dL)	8.7**	—	—
PbBn (μg Pb/g bone mineral)	1.8	1.3	0.8
PbB (μg Pb/dL)	0.2	1.8	0.3

Δ*R*^2^ for exposure only. Analyses controlled for age, alcohol, smoking, and ESs.

* *p* < 0.08

** *p* < 0.03.

**Table 3:** Unique variance (%) of CPT in the finger explained by IBL metrics with increasing criterion blood lead levels.

Variable	CPT_2000_	CPT_250_	CPT_5_
IBL	3.9*	0.4	0.0
IBL20	5.8**	1.0	0.1
IBL30	7.8#	1.8	0.2
IBL40	10.8##	2.7	0.5
IBL50	14.4##	3.7	0.6
IBL60	23.3##	10.1#	1.7

Δ*R*^2^ for exposure only. Analyses controlled for age, alcohol, smoking, and ESs.

* *p* < 0.08

** *p* < 0.03

# *p* < 0.02

## *p* < 0.005.
